# Synthesis of Qualitative Evidence on Malaria in Pregnancy, 2005–2022: A Systematic Review

**DOI:** 10.3390/tropicalmed8040235

**Published:** 2023-04-20

**Authors:** Jaiberth Antonio Cardona-Arias

**Affiliations:** Research Group Salud y Comunidad—César Uribe Piedrahita, School of Microbiology, Universidad de Antioquia UdeA, Calle 70 No. 52-21, Medellin 050010, Colombia; jaiberth.cardona@udea.edu.co

**Keywords:** malaria, paludism, qualitative research, ethnography, grounded theory, participatory action research, systematic review, meta-synthesis

## Abstract

Qualitative research on malaria in pregnancy (MiP) is incipient, therefore its contextual, experiential and symbolic associated factors are unknown. This study systematizes the qualitative research on MiP, describes knowledge, perceptions and behaviors about MiP, and compiles individual, socioeconomic, cultural and health system determinants of MiP through a meta-synthesis in 10 databases. A total of 48 studies were included with 2600 pregnant women, 1300 healthcare workers, and 2200 relatives or community members. Extensive knowledge was demonstrated on ITN and case management, but it was lacking on SP-IPTp, risks and consequences of MiP. Attitudes were negative towards ANC and MiP prevention. There were high trustfulness scores and preference for traditional medicine and distrust in the safety of drugs. The main determinants of the Health System were rationing, copayments, delay in payment to clinics, high out-of-pocket expenses, shortage, low workforce and work overload, shortcomings in care quality, low knowledges of healthcare workers on MiP and negative attitude in care. The socioeconomic and cultural determinants were poverty and low educational level of pregnant women, distance to the hospital, patriarchal–sexist gender roles, and predominance of local conceptions on maternal–fetal–neonatal health. The meta-synthesis demonstrates the difficulty to detect MiP determinants and the importance of performed qualitative research before implementing MiP strategies to understand the multidimensionality of the disease.

## 1. Introduction

Malaria in pregnancy (MiP) can progress to placental and congenital malaria and increase the risk of anemia, maternal death, miscarriage, stillbirth, retarded fetal growth, and low birth weight [1]. World Health Organization (WHO) identifies pregnant women as a high-risk group, but WHO does not present epidemiological data on MiP [2]; however, several systematic reviews demonstrate its high frequency. In Ghana, 36 investigations between 1994 and 2019 reported prevalence from 5% to 60%, higher in adolescents and young people, with decreasing trend in urban areas [3]. In India, the systematization of 16 studies reported a combined prevalence of 11.4% [4]; in Colombia, it was 16.7% based on 14 studies [5]; and in Sub-Saharan Africa, the pooled prevalence of 35 studies was 26.1% [6].

WHO strategies for malaria include prevention based on antimalarial drugs and vector control, rapid and effective case management, and elimination and surveillance [7]. These strategies demonstrate several issues that justify the need to carry out a systematic review of the qualitative evidence on MiP: (i) despite the implementation of WHO strategies, the numbers of MiP cases are high as a consequence of low-studied determinants; (ii) the strategies are oriented by biomedical perspectives, without specifying the importance of contextual aspects (socioeconomic, cultural or political determinants); (iii) the importance of the sociocultural dimension of malaria (illness and sickness) in preventive strategies, management of case and surveillance is not explicitly emphasized; (iv) the success or effectiveness of the programs is limited to surveillance and the factors of the popular heritage are not alluded to, which is important to guarantee the acceptability, use and adherence to different control actions. In general, qualitative approaches are not included.

Qualitative studies describe, understand, and explain popular concepts; study experiences, meanings and behaviors that cannot be reduced to numerical variables; focus the hermeneutic or phenomenological analysis of discourse and sociocultural situations; favor the subjective and intersubjective dimensions of social reality [8,9]. Health qualitative research addresses experiences and behaviors related to resistance or adherence to recommendations of healthcare workers (HWs), social representations of health, community responses to health care, social determinants of disease, and explanatory models of the health–disease process from the popular perspective [10,11,12].

Despite the relevance of qualitative research in health, in malaria, biomedical-positivist approach predominates. There are few systematic reviews of the qualitative evidence; some address malaria in general [13], barriers for treatment and prevention [14], response to febrile syndromes [15], and interventions to accelerate elimination [16], and in others qualitative studies are a secondary topic [17,18,19]. In MiP, only two systematic reviews of qualitative studies are available; the first systematized research on social and cultural factors affecting the uptake of MiP interventions in Africa up to 2010 [20], and the second is a meta-ethnography on motivators and demotivators for accessing MiP interventions in sub-Saharan Africa [21]. There are other reviews that include qualitative studies, but some are not systematic [22], and in others qualitative evidence is assumed as a complementary component of quantitative data [23].

This context shows the need to carry out a synthesis of the qualitative evidence on MiP with the broad approach of Cochrane; since the available reviews are not updated, they focus on a specific intervention, and it is unknown whether there is qualitative research outside of Africa. Reviews with broad Cochrane approach include the goals to seek to summarize the scientific production in a topic, update a field of knowledge, identify the main topics or gaps in research, define future lines of work, and other uses [24].

This research had the following objectives: (i) systematize the main findings of qualitative research on MiP worldwide, (ii) describe the knowledge, perceptions and behaviors related to the prevention and care of MiP, and (iii) comprehend socioeconomic, cultural and health system determinants of MiP control.

## 2. Materials and Methods

### 2.1. Study Type

A systematic review and meta-synthesis of qualitative studies applying PRISMA (preferred reporting items for systematic reviews and meta-analyses) guide were conducted. These studies relate dispersed qualitative evidence of a wide range of participants and descriptions; make a combined interpretation of the primary studies, transcending the findings of each individual investigation (the whole is greater than the sum of its parts) [25,26]. In health, meta-synthesis is useful to refine issues from the perspective of stakeholders (patients, relatives, HWs, decision makers or policy makers); increase knowledge of the disease in its natural dimension; know the interpretations and perceptions of patients about the disease and its determinants; identify the relevance, acceptability and usefulness of intervention in the community. The synthesis and systematic integration of qualitative evidence facilitates the transfer of knowledge to improve medical care and provides evidence for the development, implementation and evaluation of health interventions [25,26,27,28,29].

These studies make it possible to improve the understanding of the meanings, experiences and perspectives of the participants on the health–disease process, both in depth (due to the qualitative approach) and in breadth (due to the integration of studies with different participants and healthcare contexts) [29]; therefore, they improve the viability and acceptability of health policy decisions; they propose new theories, methods and practices in health; explain, interpret and apply the results derived from different initiatives for the prevention, treatment and control of diseases [24,25].

### 2.2. PICo (Population or Problem, Interest and Context) Question

The population consisted of pregnant women, relatives, HWs and community members recruited in qualitative studies on MiP.

The interest were the knowledge, perceptions, meanings, experiences and determinants of MiP, using categories, their properties (characteristics of a category, which define it and provide it with meaning) and dimensions (inter-subject variations for a property). A category is a concept or cluster of concepts about the attributes of a sociocultural reality described from the point of view of the subject’s experiences [30].

The context was limited to health care institutions and communities from malaria endemic areas of studied populations.

### 2.3. Search Protocol and Study Selection

#### 2.3.1. Data Source and Searches

The search terms were chosen by consulting MeSH (Medical Subject Headings), DeCS (in Spanish Descriptores en Ciencias de la Salud in Spanish) and performing a comprehensive pearl growing [31], obtaining three terms for infection (malaria OR plasmodium OR paludism), four for MiP (pregnancy OR gestation OR placenta OR congenital) and eight for the qualitative approach (qualitative OR hermeneutic OR ethnographies/ethnography OR grounded theory OR community-based participatory research/community-based research OR participatory research/participatory action research OR cultural anthropology OR ethnopsychology). The combination of these terms generated eight strategies applied in PubMed, OVID EMCare, Scielo, LILACS, Scopus, Web of Science, Science-Direct, Jstor, Campbell Collaboration/Cochrane Library, HAPI and Google Scholar. The Search syntax applied to database were:

PubMed and OVID EMCare: ((Malaria[Title/Abstract] OR paludism[Title/Abstract] OR plasmodium[Title/Abstract]) AND (Pregnancy[Title/Abstract] OR Gestation[Title/Abstract] OR Placenta[Title/Abstract] OR Congenital[Title/Abstract])) AND (qualitative[Title/Abstract]). Scielo, LILLACS and Jstor: (ab:(Malaria OR plasmodium OR paludism)) AND (ab:((Pregnancy OR Gestation OR Placenta OR Congenital)) AND (ab:(qualitative)). Scopus: TITLE-ABS-KEY (((malaria OR paludism OR plasmodium) AND (pregnancy OR gestation OR placenta OR congenital) AND (qualitative))). Web of Science: ((Malaria OR paludism OR plasmodium) AND (Pregnancy OR Gestation OR Placenta OR Congenital) AND (qualitative)) (Abstract). Science-Direct: (Title, abstract, keywords: (malaria OR plasmodium OR paludism) AND (Pregnancy OR Gestation OR Placenta OR Congenital) AND (qualitative)). Cochrane Library: (Malaria OR plasmodium OR paludism in Title Abstract Keyword) AND (Pregnancy OR Gestation OR Placenta OR Congenital in Title Abstract Keyword) AND (qualitative in Title Abstract Keyword). HAPI: Title: Malaria OR plasmodium OR paludism (and) Title: Pregnancy OR Gestation OR Placenta OR Congenital (and) Title: qualitative. Google Scholar: allintitle: (Malaria OR paludism OR plasmodium) AND (Pregnancy OR Gestation OR Placenta OR Congenital) AND (qualitative).

In the remaining 7 syntaxes, only the last word is changed for the 7 additional terms for qualitative methods. These syntaxes were also applied in Spanish and Portuguese. This process was complemented with the application of additional searches with the terms maternity, focus group, interview, perception, belief and attitude, and with the truncation* for the terms pregnan* ethnograph* and anthropolog*. Additionally, a manual search was performed in the references of previously published systematic reviews of qualitative studies [20,21,22,23]. The search for studies did not have time restrictions, the last update was carried out in December 2022 and the first study identified is from 2005.

#### 2.3.2. Eligibility Criteria

To the records identified with all the search strategies, the first inclusion criterion was applied: include the search terms in the title or abstract. Subsequently, the manuscripts were saved in a common source in Zotero to eliminate duplicates, and to apply the following inclusion criteria: studies on MiP as the main topic, qualitative research (quantitative or mixed studies were eliminated), and original investigation (reviews, editorials, essays were excluded).

Exclusion criteria: three manuscripts were excluded because, despite indicating in their abstract that they included qualitative themes on MiP, they did not include results on this topic.

### 2.4. Data Extraction, Quality Assessment and Reproducibility

The following variables were extracted of the included studies: title, authors, year of publication, year of realization (execution) of the study, place of study, central topic, objective, number and characteristics of the study subjects, categories with its properties and dimensions, conclusions, and items of methodological quality. The methodological rigor was evaluated with Standards for Reporting Qualitative Research (SRQR) guide [27], which has an applicable design to all qualitative methods. To ensure reproducibility, the researcher applied the search and selection protocol at two different times.

### 2.5. Data Analysis

The percentage of quality criteria fulfilled by each study was determined. To this, an analysis by item (number of studies that applied each criterion) was added. A qualitative synthesis of the main topics and populations of each study was carried out. The countries and study populations were mapped. Five topics were identified, an exhaustive description of each one was recorded based on the categories and subcategories identified (knowledge, perceptions, behaviors and individual, socioeconomic, cultural and health system determinants of MiP); in each subcategory, a description of its properties and dimensions was recorded. A meta-synthesis was carried out to explain the relationships between the topics, categories and subcategories of the included studies, and a list of recommendations was derived of the synthesis of the available qualitative evidence.

## 3. Results

**Figure 1:**
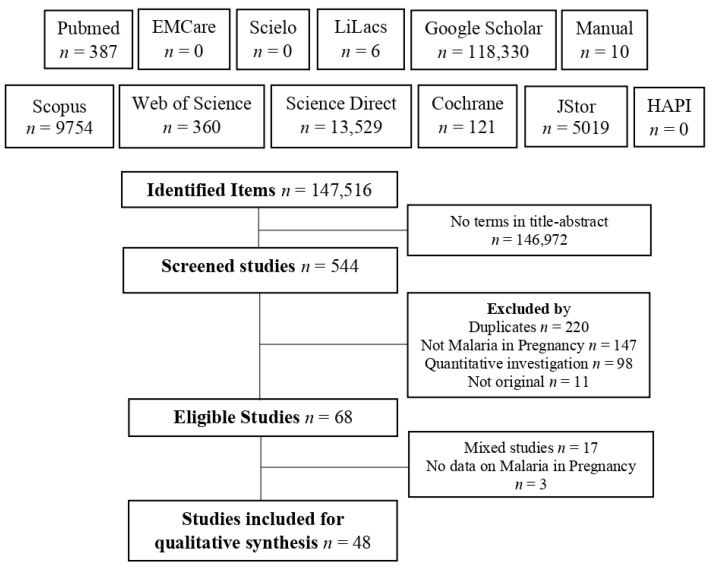
Flowchart of search and selection of studies.

Applying all the searches generated 147,516 results of which only 544 included the terms in the title or abstract; 48 studies met the eligibility criteria (Figure 1).

### 3.1. Description of Included Studies and Methodological Quality

In the 48 included studies, 96% used in-depth interviews, 63% employed focus group discussion, and less than 10% applied observation (in waiting rooms, ANC or communities), participatory techniques (lists and free ratings, rankings, mapping) or informal conversations. The most frequent topics were SP-IPTp (intermittent preventive treatment in pregnancy with sulfadoxine-pyrimethamine) (54%, *n* = 26), MiP (27%, *n* = 13), ITN (19%, *n* = 9), case detection and management (15%, *n* = 7), ANC (antenatal care) (8%, *n* = 4) and experiences in MiP research (Table 1).

**Figure 2:**
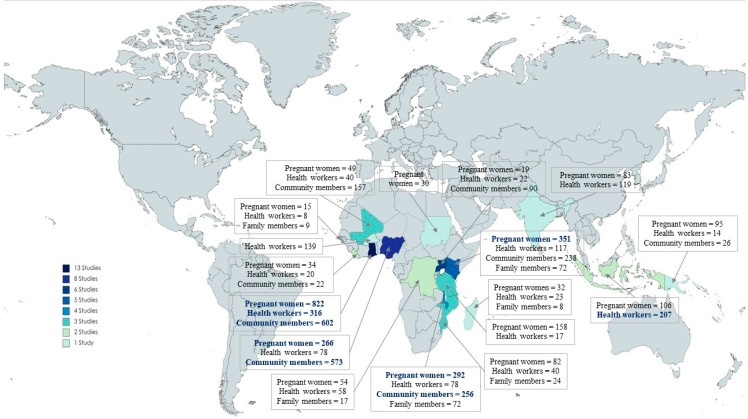
Number of studies according to country and investigated populations.

The population was approximately 2600 pregnant women, 1300 HWs, 2200 relatives, and community members. Only four studies were conducted outside of Africa; the countries with the most studies were Ghana (27%, *n* = 13), Nigeria (17%, *n* = 8), Uganda (13%, *n* = 6) and Kenya (10%, *n* = 5) (Figure 2).

**Figure 3:**
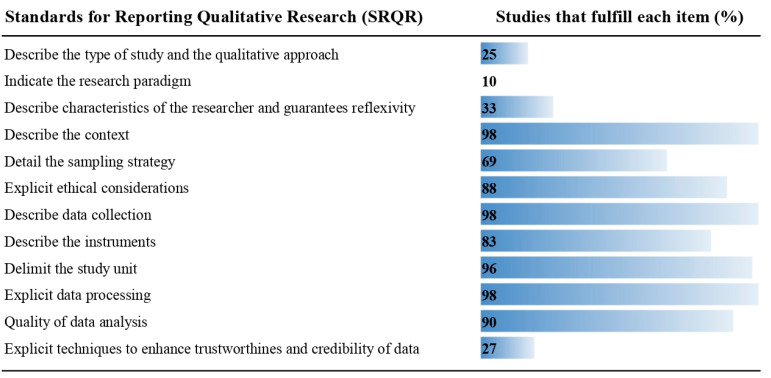
Assessment of the methodological quality of the included studies, according to Standards for Reporting Qualitative Research (SRQR) guide.

Regarding methodological rigor, the studies met between 25% and 92% of the criteria of the SRQR guide (Table 1); the least applied criteria were the following: Indicate the research paradigm (10%), Describe the type of study and the qualitative approach (25%); Explicit techniques to enhance trustworthiness and credibility of data (27%); and Describe characteristics of the researcher and guarantees reflexivity (33%) (Figure 3). Table 2 summarizes the main categories and subcategories on ANC, MiP and three WHO strategies.

### 3.2. Antenatal Care

#### 3.2.1. Positive Perceptions

Pregnant women, relatives and community members have favorable perceptions of ANC. The motivations for attending ANC include receiving health education, preventing diseases, knowing the health status of the mother/child, performing laboratory tests of maternal–fetal monitoring and screening of diseases, administering timely treatment of alterations, receiving drugs or vitamins, and avoiding complications through attention of physicians, specialists, nurses, and community health staff in an organized and simultaneous manner [42,68].

Pregnant women highlight the importance of courtesy and patience of HWs, their trust in biomedicine and HWs (women do not know the purpose of the drugs or blood tests, but they accept them), the importance of including home visits by HWs mainly in dispersed rural areas, and making services free to avoid out-of-pocket costs [42,68]. Promoting these issues guarantees high acceptability, use and continuity of ANC.

#### 3.2.2. Experiences That Prevent the Use of ANC

Negative experiences and health costs determine the healthcare seeking. Economic costs function as the main barrier to access. These include transportation to clinics, direct expenses for travel and food, loss of time due to the need to attend appointments (added to long waiting times or delays in care), delays in domestic responsibilities and work losses [41]. These expenses are increased by distance to hospital, shortages that increase the patient’s expenses for the purchase of utensils or drugs, and absence of community work or home visits by health personnel [35,42]. Costs of referral services, user fees at ANC clinics, unofficial payment and penalties by private service providers when they do not comply with service hours were also described [41].

Negative perceptions of ANC are determined by sociocultural, institutional, and individual factors. In sociocultural determinants, gender roles and worldviews of rural territories explain the low value conferred to healthcare of the mother/child, and scarce support of relatives, community members or HWs to receive ANC in a timely manner [68]. In institutional determinants, the discourteous and impatient attitude of some HWs, especially in rural areas, increases the frequency of consultations by midwives [33,35,42]. In individual determinants, some pregnant women or their relatives make the decision to refuse to take medication, skip appointments or start ANC late because they do not value its importance or have a more favorable perception of traditional medicines [68].

### 3.3. Malaria in Pregnancy

#### 3.3.1. Knowledge Attitudes (Perceptions) and Practices

Pregnant women, relatives, community health staff and community members use different terms to define malaria, which overlap with biomedical definitions, but with variations in knowledge regarding etiology, severity, risk groups, and MiP consequences [39,45,50,52,76,79]. MiP is conceived as a cause of poor health in pregnant women. There is a good level of knowledge on some risks, but without differentiating them from other febrile syndromes or pregnancy symptoms [33,35,50,51,64]. For pregnant women and their families, initial symptoms of MiP are perceived as normal, and they assign low importance to its control [39,45,50,52,64,76,79].

Pregnant women cite mosquitoes and other incorrect modes of malaria transmission, which was also reported by some HWs. They are aware of the ITN-based prevention, but few know of other preventive measures, and they prefer traditional strategies instead [36,52,64,65,79]. Regarding treatment, a minority of pregnant women and the majority of HWs were aware of malaria treatments, although they also included unproven methods [64].

Regarding MiP consequences, pregnant women reported weakness, anemia, abortion, anemia, and low birth weight, but these outcomes are reported as intrinsic risks of pregnancy and they are not specific of MiP [35,45]. This result is different in multigravidas and women with previous episodes of malaria; based on their own experience, they have a better understanding of MiP consequences [79].

There are positive perceptions regarding MiP care attributable to the courtesy of HWs and trust in biomedicine. Pregnant women and their relatives reported positive attitudes in relation with checking for anemia and seeking treatment when the pregnant woman feels sick; but simultaneously, there is a low perception of risk of MiP, especially in primigravids and adolescents [35,65]. This converges with attitudes of carelessness or indifference in some pregnant women towards the use of preventive methods [65], although in other contexts, the importance of ITN-based prevention is highlighted [79]. Additionally, favorable perceptions of the ITN are based on the comfort of sleeping without bites, protecting several people and preventing the use of drugs. They highlighted barriers to access related to the cost of acquiring ITN and bad attitude of the HW at its delivery [50,52,64].

The attitudes towards treatment were negative because pregnant women express concern about pharmacological therapies for MiP, citing potential harm to the baby [55,64]. There is a better perception of traditional health system, with trust in the safety of homemade concoctions and recommendations from traditional healers or physicians, which is fostered by family and community initiatives [48,55].

Knowledge and attitudes are associated with behaviors on MiP prevention and treatment. Drugs and biomedicine are trusted in cases of advanced disease. Use of traditional medicine is most frequent in a sequence that includes self-care based on knowledge or previous experiences with MiP, consultation with relatives, consumption of home remedies and concoctions, consultation with healers, self-medication with biomedical options and purchase to private vendors, and ultimately, the diagnosis and treatment of MiP is sought in the clinic [33,36,48,50,51,55,79].

The use of preventive methods is determined by availability, safety, and convenience. Pregnant women use at least one method, although irregularly and in conjunction with traditional methods such as burning leaves or rubbing oils on the body [50,64]. Traditional medicine also predominates in treatment; the use of herbal and spiritual remedies is frequent, while adherence to antimalarials is low. This situation has been explained with the following reasons: trust in traditional system or advice of relatives, distance to clinics and drug dispensaries, domestic workload of pregnant women, proximity to midwives and healers, believing that most symptoms of MiP are normal and inherent to pregnancy, and negative perceptions of drugs due to their adverse effects [50,55].

#### 3.3.2. Determinants of Prevention and Management

Local worldviews explain the trust in traditional medicine, determine the conception of the health–disease process, define the time and place for seeking health care and the preventive or therapeutic strategies used [35]. These determinants are feedbacked by barriers to access to biomedicine, the social role of women, the preference of male partners for traditional medicine and socioeconomic factors such as low education (which hinders access and use of information on MiP), poverty (which hinders access to the educational system, health services, and increases risk of becoming sick) and economic dependency (it prevents pregnant women from being autonomous in decisions). These factors are obstacles to accepting, using, and accessing biomedical preventive and therapeutic strategies [33,36,48,50,55,65,79].

The cultural determinants (gender roles, community conceptions of health, traditional medicine, etc.) overlap with the socio-economic factors (low education, monetary poverty, etc.), and both types of determination are reinforced with problems in the health systems that hinder or decrease the effectiveness of the prevention and management of MiP. The health system determinants include few financial resources, corruption, lack of trust in the health care establishment; shortages in medicines, diagnostic tests and other supplies; delays in national institute of health reimbursement to clinics, requirement of copayments, rationing of drugs, and formulation of medicines for women to purchase in private pharmacies [65,68].

These determinants cause negative experiences in health care of the pregnant women, which produces meanings and behaviors that prevent the patients from ANC and MiP programs and at the same time reinforce the preference for traditional medicine [36,79].

### 3.4. Insecticide Treated Net

A high level of knowledge, awareness and positive attitudes related to the benefits of ITN were determined; its effectiveness is highlighted to prevent the disease in pregnant women or several relatives and to avoid bites of various mosquitoes that are vectors of different diseases [33,40,46,51,59,60,76]. In relation to the insecticides used, some participants are sure of their safety since they do not affect the pregnancy [51,59,60,76], while others indicate dangerous effects on the pregnancy and the fetus [33,40]. In some areas, high degree of awareness coexists with some negative attitudes based on the discomfort caused by the heat and the smell of ITN, irritating effects on the skin or mucous membranes, difficulties hanging the net, avoiding holes in it, or applying retreatment [59,60,66].

The higher knowledge and attitudes and the most constant and correct use were reported in women who used the ITN since before becoming pregnant, mothers of young children, people with greater awareness of MiP risks, and pregnant women who attended ANC services and were cared for by HWs with better attitudes. Negative attitudes were greatest among pregnant women who experienced irritating effects of ITNs, and whose families or communities prefer traditional methods to repel mosquitoes [51,66,76].

The acceptability and use of ITN are explained by cultural and health system factors such as trust in traditional pest control methods and erroneous beliefs about ITN chemical products, ANC services or biomedicine [33,40,51,66,76]. Family and gender roles are also included, power relations between husbands, decision-making at home, lack of interest of the husband in MiP prevention, type of medicine that should be used, and situations where biomedicine is accepted. Community norms, beliefs and practices define the perception of MiP risk; for example, the belief that adolescents and primigravid women have a low risk of contracting malaria leads to the conclusion that for this reason, it is not necessary to prevent it. In addition, in some contexts, magical–religious visions of health predominate [33,40,51,60,66,76].

In the socioeconomic and health system determinants, the main barriers to the use of ITN were the high cost [33,40,41,51,59,66], logistical and financial difficulties for free distribution [59,76], housing type [60,66] or having to stay out of home late for work reasons [66]. Health service determinants were shortages or low availability of ANC services, problems in the supply chain [46,47,51,78], problems of governance and financing, low health workforce [33,40,47,78], HWs with low training on MiP [47,66,76,78], poor perception of service quality, and HW malpractice [51,76].

### 3.5. Intermittent Preventive Treatment in Pregnancy with Sulfadoxine-Pyrimethamine

#### 3.5.1. Knowledge Attitudes and Practices

Pregnant women and ANC personnel know that SP is the recommended drug for IPTp; HWs (except midwives) highlight SP as an effective drug to prevent and cure malaria [32,34,35,76]; however, the most recurrent finding in pregnant women is poor knowledge about the purpose of drugs prescribed during pregnancy. They do not have information to judge the safety or efficacy of this intervention, and there is low awareness of the importance of this strategy [37,39,51,67,76]. In some countries, pregnant women do not associate malaria with SP-IPTp, they do not know why they are prescribed different medications and vitamins during pregnancy, and when they took SP-IPTp, they thought it was a treatment and not a preventive strategy [42,46,68,76].

Pregnant women are dependent on HWs, and ANC provider becomes a key actor for the access and use SP-IPTp [39,76], but some HWs do not know the trimester of pregnancy to start it, number of doses, implementation guidelines, adverse effects or benefits for mother/child [37,38,43,47]. In other cases, HWs do not have written guidelines or instructions on the administration of the treatment, or the guidelines they use are inconsistent or different to WHO recommendations, evidencing a lack of training, supervision, and knowledge of the protocols in the clinics [53,58,78].

Pregnant women accept drugs due to their effectiveness in MiP control, but simultaneously they report concern, distrust or anxiety due to its side effects because pregnant women believe that SP can weaken them, alter maternal–fetal health, cause abortions and fetal anomalies [32,33,34,35,39]. Some pregnant women believe that its use generates drug resistance, and others consider unnecessary to seek care for a disease they do not yet have [34,35,44].

Some pregnant women accept SP-IPTp and ANC if this practice is encouraged by HWs. Relationship with providers is also important to influence acceptability and use of SP-IPTp [57,76]. Positive attitudes are more recurrent in pregnant women who have received good-quality ANC services, with a polite and patient attitude of HWs, and good quality of information provided by physician or nurses [42,43,57]. Other authors also highlight the importance of building trust with the communities for the delivery and use of SP-IPTp, the integration between the community health system (promotion of socially integrated practices) and knowing previous experiences of women, relatives and friends [69,76].

Negative attitudes were recorded in nurses and providers due to supposed risks for the mother and the baby [32,62]. In physicians, this intervention is well perceived for its efficacy, but there are perceptions of skepticism and distrust regarding the compliance of direct observation therapy due to the scarcity of clean water and vessels in some ANC clinics, aversion to drugs in some pregnant women, late admission to ANC, and misconceptions about its safety [32,38,57,73].

The following practices of pregnant women were highlighted: they throw away SP tablets after leaving the clinic [32], they are totally dependent on HWs to obtain drugs [39], women display low adherence to directly observed therapy, high proportion of providers dispense SP to take at home assuming pregnant women would take their drugs [43,49], self-medication is high throughout pharmacies [44], many do not comply with the treatment scheme, citing previous side effects or the fact of not being sick [46,49,53]. Nurses prefer to provide directly observed therapy because they do not trust pregnant women to take the pills; physicians are skeptical about the compliance of therapy due to infrastructure problems in the clinic, and consider that the problems of use of SP-IPTp are not related to demand but to supply, so it is crucial to ensure a regular supply of SP [32,53,57,72].

#### 3.5.2. Determinants of Acceptability

In the individual determinants, personal decision not to attend ANC appointments or enter late [37,71], limited understanding of the SP-IPTp scheme (questions about the time it should it be started, SP dosage, and duration of intervention were reported) [37,44], refusal to take SP mainly because of its possible side effects on maternal/fetal health [43,51,53,71], resistance or rejection of drug-based preventive measures (what is the reason for taking medicines without being sick?), low perception of MiP risk, low awareness of the importance of SP-IPTp [44,67], and cultural determinants of individual decisions such as the type of family support, roles of the pregnant woman and family decision-making related to health care [44,71,75].

Most studies highlight health system problems as the main determinants of the acceptability and use of SP-IPTp: (i) problems in the organization of ANC services or late admission due to lack of personnel [38,53,56,67,71,75]; (ii) quality of health care in relation to the interaction with HWs, perception of their competencies, poor attitudes, corruption or malpractice of health staff [51], poor strategies of HWs to administer SP [71], unreliable supply of free SP [38], poor service planning [38], long waiting times [51] and scarcity of clean and safe water [38,43]; (iii) inadequate information distribution to pregnant women [71], HWs without motivation, training, education and supervision on this strategy [37,38,44,47,78], complex policy guidelines or lack of protocols of implementation [47,75]; (iv) work overload and insufficient time for counseling [53,67]; (v) stock-outs [37,43,44,51,58,68,71,78], delays in national procurement [78], and problems in supply chain [78]; (vi) high out-of-pocket health expenses and financial limitations [38,51,56,71], delays in reimbursement from the national health institute increasing co-payments, rationing or purchase of drugs in private pharmacies [68], and (vii) structural problems of governance, financing and human resources of health systems [47,75].

### 3.6. Detection and Treatment of Cases

Positive perception of the diagnosis and the efficacy of the antimalarials predominated, and only negative attitudes were registered in relation to the adverse effects of these drugs. Detection and treatment are acceptable because these strategies help to have a healthy pregnancy. Pregnant women like to be tested at each ANC appointments, achieve timely detection and treatment of infections, reduce unnecessary exposure to drugs during pregnancy or the development of resistance to them [42,46,51,54,62,74]. Despite this, there is low information on the safety of antimalarials in pregnancy, which generates a negative attitude and low adherence in some pregnant women [46,51,54].

Some HWs distrust the accuracy of rapid diagnostic tests and the safety of some drugs. Other barriers are distance to clinic, low availability of diagnostic tests and drugs [54,61,62,70]. The adherence can be affected by the quality of the service (interaction with HW, perception of their skills and provider malpractice), and distrust of providers related to pregnant women about adherence to multi-day regimens when it must be consumed outside of institutions [42,46,51,54,74].

Socioeconomic barriers are related to the distance to clinic, the costs for pregnant women or their families to access this strategy, which leads to the choice of taking herbal remedies or drugs bought in stores [51]. Problems with the availability of diagnostic tests and drugs that prevent or delay diagnosis are also highlighted, which leads to increased use of the traditional medical system, exacerbates negative attitudes towards this strategy and ANC, and increases self-medication [46,61,62,74].

### 3.7. Utility of Investigations in MiP

Pregnant women have a positive perception about the participation in research (especially in clinical trials) because these projects overcome barriers to ANC and MiP care; they receive drugs and undergoing diagnostic tests; HWs have better attitude and provide more information about the benefits for mother–child health [42]. Others highlight the importance of research to improve educational programs that change negative attitudes toward malaria prevention, diagnosis, and treatment seeking [54,65].

To optimize the participation of pregnant women in MiP research, it is recommended to provide evidence-based education on malaria, encourage the participation of traditional leaders in the design of the research, build community platforms for discussion and dissemination, map out communication and information errors, fears, and cultural barriers, capitalize previous investigative experiences in the community, and facilitate transportation to the clinic for pregnant women who do not accept home care or prefer hospital sampling [49,63,77].

**Figure 4:**
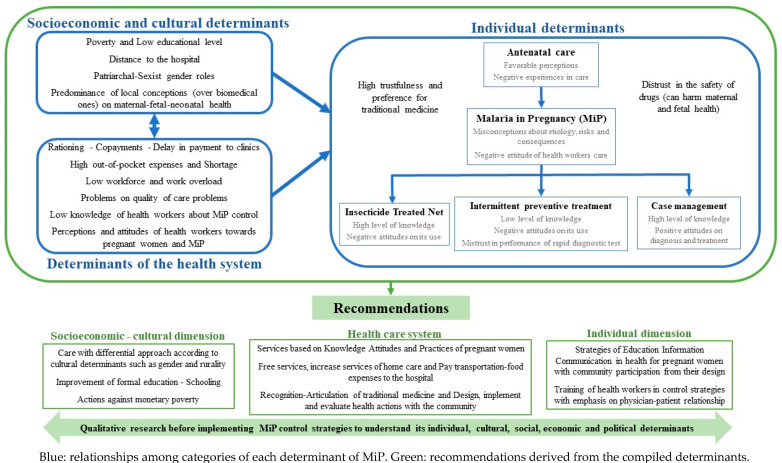
Meta-synthesis of socioeconomic, cultural, health system and individual determinants of MiP control.

Figure 4 lists the main themes and categories, highlighting five aspects: (i) individual determinants of ANC and prevention, diagnosis and treatment of MiP, (ii) socioeconomic and cultural determinants that influence the results of the individual dimension, (iii) determinants of the system health, which are reinforced with the socioeconomic and cultural determinants, and also impact the individual dimension, (iv) recommendations for malaria control programs based on each type of determinant, and (v) highlight of the importance of qualitative research as a transversal component to all the dimensions and determinants.

## 4. Discussion

### 4.1. Main Findings

In this review, 48 qualitative studies were included with 2600 pregnant women, 1300 HWs, and 2200 relatives and community members; the most frequent topics were the meanings, perceptions and behaviors on MiP, SP-IPTp, ITN and case management, which is similar to a systematic review on mixed studies about MiP [80].

In the individual domain, pregnant women have a high level of knowledge about ITN and case management, but the level of knowledge is low in SP-IPTp, risks and consequences of MiP. Pregnant women, relatives and community members had negative attitudes towards ANC and MiP prevention; they had high trustfulness and preference for traditional medicine, and distrust in the safety of drugs (can harm maternal and fetal health). According to pregnant women, relatives, community staff and HWs, the main determinants of the Health System that affect MiP control include rationing, copayments, delay in payment to clinics, high out-of-pocket expenses, shortage, low workforce and work overload, poor quality of care, and low knowledge of HW about strategies of MiP control. The socioeconomic and cultural determinants are poverty and low educational level of pregnant women, distance to the hospital, patriarchal–sexist gender roles, and predominance of local conceptions on maternal–fetal–neonatal health.

### 4.2. Saturated Qualitative Evidence

The qualitative evidence of this review is consistent with that of other systematic reviews of qualitative studies on MiP in various topics that were grouped into four domains: conceptions of MiP, attitudes and behaviors regarding control strategies, health system factors that affect its prevention and management, and structural determinants. In the first group, this and other reviews recognized MiP as a risk event but pregnant women do not perceive their vulnerability to this disease. Local interpretations overlap the symptoms and consequences of MiP with other febrile syndromes or are assumed as conditions inherent to pregnancy, and there is preference for the diagnosis and treatment over prevention [20,21,80]. Attitudes and behaviors related to MiP interventions are associated with attitudes and interactions with HWs, the organization of health services, and experiences of poor health care [20,21,23]. The main health system factors that affect prevention and management actions are the training of HWs, cost and distance to health facilities, lack of health care infrastructure, and availability of medical supplies [20,21,22,23,80]. Structural determinants of MiP control include household decision-making, gender relations, and the availability of financial resources [20,21,80].

This convergence or saturation of qualitative evidence allows to infer several practical implications. First, it demonstrates the challenges of MiP control that do not require further research but should be transformed into lines of action or public policy due to their repetition in various populations. It identifies areas where quantitative evidence is not necessary due to the large number of people grouped in these reviews, where the magnitude of the problem is shown. It highlights reiterated topics in many contexts, where it is only possible to apply qualitative methods due to their impossibility of reducing numerical variables (for example, gender roles), but which are not traditionally included in control strategies focused on biomedical or epidemiological evidence.

The consistent evidence in these four domains also demonstrates that qualitative studies broaden and deepen the traditional evidence of health research, characterized by its biomedical emphasis, focused on the singular domain, and with ontological and methodological individualism, which will not allow the effective MiP control, because it does not impact the other determinants identified. To focus the health behavior individually implies committing the atomistic fallacy (false generalization about group variability based on individual information) or the psychological fallacy (explaining interindividual variability without including group variables, without analyzing the contextual or structural effect), as well as ignoring the importance of the community (as something different to sum of individuals) in their empowerment and ability to treat health problems [81,82]. Individualism can become an ideology with negative effects on patients and HW because it is partial, biased, and exploits people; for promoting a consumer medicine that exalts individualism as the only approach to subjective well-being, highlights autonomy as the only ethical principle (even superior to justice or beneficence), reduces the human being to their psychological self with neglect of the social, cultural and political dimensions, and turns physicians into healers or bodily engineers [83,84].

Clustering into the four domains also shows the importance of multilevel analyses of reality to decrease MiP burden and define the scale and temporality of the control strategies. Actions in singular level with short-term results in pregnant women or HWs. Strategies in particular level produces medium-term effects in community or families. General level implies long-term efforts and results on structural aspects of the health, economic, or cultural systems [23]. This multiple causality has been discussed in epidemiology for more than five decades, but these theoretical models have scarce application in MiP. Therefore, the results of this review are of greater importance when highlighting the multiple causation of MiP and the need to understand the subjective and intersubjective domain of reality to design and to implement control strategies [13,20,21,22,23]. These repetitive findings are also important considering that since the last century, multiple theoretical models of public health have been worked on, which highlighted the importance of addressing the social determinants of health, but in malaria, the appropriation of this approach is incipient, and in MiP, it is practically nil [85,86].

Finally, the consistency of these findings demonstrates the need to continue reducing MiP burden, but broadening the focus towards popular beliefs and attitudes about the disease, the intervention of health system factors and social, economic and cultural determinants; given that pregnant women act under their conceptions and contextual limits, a more holistic understanding of the illness and sickness dimensions is deserved, since the focus has been on the disease dimension. In this line, it is crucial to include qualitative research methods, which are well adapted to the study of structural factors at multiple levels and allow to situate phenomena within the individual–personal, family, community, cultural, social, economic and historical order [16,18,20].

### 4.3. New Qualitative Evidence of This Meta-Synthesis

**Figure 5:**
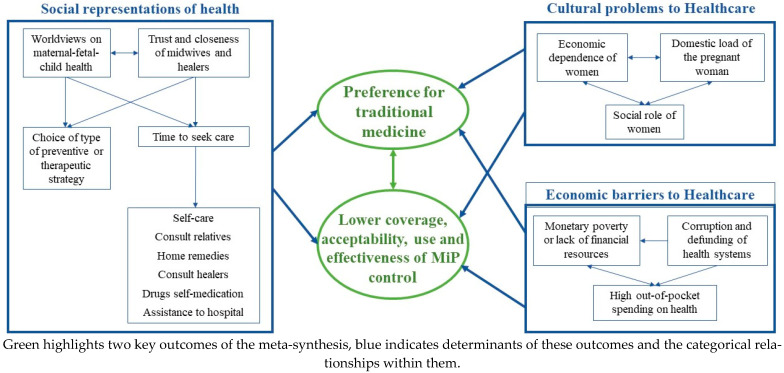
New category relationships to understand determinants of MiP control.

Given the complexity of discussing each category found in this meta-synthesis (in addition, doing so would be contradictory to qualitative research that seeks to generate explanatory and relational systems), the novel concepts of this review were grouped and related in a conceptual order that will guide the following discussion, highlighting several determinants of the preference for traditional medicine and of a low effectiveness of strategies of MiP control (Figure 5).

Trust in and preference for traditional medicine were determined, both in MiP and specific strategies such as ITN or SP-IPTp. The preference for traditional medicine leads to the WHO strategies having low coverage, acceptability and use to reduce MiP burden. This preference for traditional medicine and lower effectiveness of strategies for MiP control is determined by the social representations of the disease (a complex, multicategory dimension and with diverse relationships within it) and by cultural and economic barriers (integrated by several categories that are also related in different ways). These characteristics are context-dependent and merit the performance of original studies that show the variations of the properties and dimensions of each category in specific places as an axis for the design of action on MiP control. Low coverage in control strategies, cultural and economic barriers to access biomedicine, and social representations of health are feedbacked by different mechanisms that increasing preference for traditional medicine. The complexity of these relationships has also been described in systematic reviews of quantitative studies on malaria, in which the frequency of use of traditional medicine for the treatment ranged from 1 to 40% in various endemic sites, higher in rural areas, women, people with low educational level and ethnic minorities. In addition, the use of traditional medicine delayed the search for treatment in health centers and was explained by the lack of accessibility to biomedical health services (caused by geographical and financial barriers), faith in traditional treatment, and low perception of malaria risk [18,87]. These aspects should be included in strategies for the prevention, care, and surveillance of MiP, given that the qualitative evidence of this review and other quantitative evidence converge on these findings.

For MiP control, it is important to know in depth the social representations of health, specifically the therapeutic itineraries that in this meta-synthesis showed the hospital attendance as the last option, which has also been reported by other authors [16]. The social representations of the health–disease process are determinant for MiP since this is the knowledge that is socially constructed based on experiences and thought models received by education or social tradition; it elaborates personal and social identity, configures a system of interpretation of reality, and guides behaviors or practices. Social representations define the care-seeking, the importance attached to initial symptoms, early diagnosis, the choice of a preventive or therapeutic practice, and adherence to treatment [88,89]. As part of the symbolic universe, social representations are systems of assumptions for experiencing the self and the world; they function as a universe of meaning charged with affect; they are asemantic (they do not require scientific evidence or verification of reality), involve the entire field of experience instead of its individual parts, and they are a translation mechanism of the subjective world that underlies the health and illness behavior of patients [90,91].

Regarding the economic barriers to access biomedical health services, high out-of-pocket expenses, corruption in the use of health resources, lack of financing of health systems, poverty and lack of economic resources of families are highlighted. These factors are related; they reinforce and exacerbate the problem of MiP control due to its dependence on structural aspects, generally not addressed in health care-related actions. The economic burden of malaria treatment is high for families, especially considering that the most affected populations are poor. Some reports indicate that 70% of patients incur costs to seek medical attention, but in quantitative studies, this has only been associated with living far from hospital, while in this meta-synthesis, the mechanisms that generate and make this situation more complex are expanded upon. It has also been reported that 66% of patients have productivity losses of five working days during an episode of malaria for them or their child; despite having free treatments, households report economic losses attributable to the disease [92]. This is serious considering that this disease generally occurs in poor populations, exacerbating the vicious circle of malaria–poverty and its multiple mechanisms of interaction. For example, malaria implies a high economic burden for the patient, the health system, and society due to productivity losses. This exacerbates the poverty of those affected on account of the resources that must be allocated to their treatment and the income they lose due to attending to their illness. At the same time, poverty acts as an obstacle to proper care of the disease, increasing the probability of advancing to severe stages, which in turn causes the patient to incur additional costs [93,94,95]. This vicious circle can only be broken by articulating a malaria control policy with a social–economic policy against poverty [15,17,18,95].

Cultural barriers related to the roles of the pregnant woman, her domestic burden and economic dependence were highlighted, as well as the issues that hinder or delay biomedical care and reinvigorate the importance of traditional medicine. This demonstrates the importance of addressing MiP with a culturally sensitive approach to care (adjusted to social and cultural contexts) as an ethical–political imperative to overcome intercultural barriers to healthcare [14,15,17,96]. It also demonstrates the urgency of addressing MiP with a gender approach, which, according to the WHO, corresponds to social constructions that define the roles, characteristics, activities, expectations, and opportunities that are considered appropriate for men, women, children, and people with non-binary identities, in a certain sociocultural context. The need to address MiP with this approach lies in the fact that gender is a determinant of health and inequities; of the risks, behaviors and responses of the health system to the disease; of the employment and working conditions; of the access to services and healthcare pathways, among other central factors for healthcare management. The problems of pregnant women included in this meta-synthesis show the importance of increasing women’s empowerment, equal participation in decisions about their health and general well-being as maxims of the gender approach applicable to MiP control [97].

### 4.4. Limitations and Strengths

Few studies reported trustworthiness, credibility and reflexivity, showing the need to improve qualitative methods in MiP. Several studies indicated worse results regarding knowledge, attitudes, and practices in adolescents or primigravid women, but did not delve into the explanatory factors of such experiences. Most of the studies were descriptive and focused on content analysis, so few advanced to explanatory or theoretical phase. Some categories were not dense in their properties and dimensions, despite their relevance, such as the ways of building trust with communities, the integration of the health system with the community, and the promotion of socially integrated healthy practices among other factors that are approached with a predominance of the etic perspective (or theoretical framework of the researchers, outsider’s perspective); however, it is not deepened from the emic perspective (insider’s perspective).

The results of this research converge with those of a systematic review of mixed studies on MiP, but the mixed studies prioritized their quantitative results, reporting the qualitative component in a smaller number of people. Their findings were less dense-deep in the categories, describing MiP determinants in a marginal succinct manner, not explaining the processes of social determination of MiP nor the feedback mechanisms of socioeconomic, health system, cultural and individual determinants, and not specifying recommendations consistent with each type of MiP determinant. However, both systematic reviews should be analyzed based on their complementarity (consistency of some categories, importance of multilevel analysis, need to broaden the complexity of actions against MiP to achieving WHO goals) and sequentiality (the synthesis of mixed studies focused quantitative results and identified some qualitative categories, but without delving into their properties, dimensions and relationships, these issues constitute the core of this synthesis of qualitative evidence). In addition, the scarcity of research on MiP, its concentration in Africa, and the targeting in WHO strategies could support the convergence of findings of the 48 qualitative studies and the 21 mixed studies. The main strengths of the study include the pooling of all the qualitative evidence on MiP in the world, with a broad Cochrane approach, without being restricted to representations of the disease or a control strategy. This made it possible to relate broad but scattered evidence based on the perspective of those affected in the natural or daily contexts where MiP occurs. Evidence and explanatory theory on MiP and its determinants were generated, which helps to improve medical care and control programs based on the multidimensional and multilevel character (singular, particular and general) of the problem. This discloses areas of low intervention in MiP strategies, which, however, are decisive to achieve the control and elimination objectives. Meta-synthesis reveals ethical problems of the physician–patient relationship, sociopolitical problems of the social determinants of health, the urgency of incorporating the gender perspective, the need to articulate biomedicine and traditional medicine and to specify control actions focused on the illness and sickness dimensions since current knowledge focuses on disease.

## 5. Conclusions

The meta-synthesis evidences the multiple relationships between knowledge, perceptions and behaviors related to the control of MiP while showing the complexity of intervening with this health problem given that actions are required that impact individual determinants (education information communication in health for pregnant women and HWs with community participation from their design; training of HWs in control strategies and physician–patient relationship), those of the health system (services based on knowledge attitudes and practices of pregnant women and HWs, free services, increase in services of home care and payment of transportation/food expenses to the hospital, recognition/articulation of traditional medicine, and design, implementation and evaluation of health actions with the community) and those of the economic, social and cultural domain (care with differential approach according to cultural determinants such as gender and rurality, improved schooling, and actions against monetary poverty). Qualitative research should be conducted before implementing MiP control strategies to increase their coverage, acceptability and use, to understand the multiple determinants that must be addressed to meet elimination goals and to improve the efficiency and equity in the use of resources for malaria programs.

## Figures and Tables

**Table 1:** Description of included studies by year, main topic, population, and methodological quality score.

Author—Country	Year *	Main Topic	Population	SRQR Score
Mubyazi—TZA [32]	2005 (2004)	Knowledge attitudes and practices (KAP) on malaria, emphasis on IPTp	Health managers, health services personnel in clinics, antenatal (ANC) service providers, pregnant women	67 ^a1^
Mbonye—UGA [33]	2006 (2002–2003)	Perceptions and barriers in the use of ITN	Adolescent (10–19 years), young (20–29 years) and adult (30–49 years) women, opinion leaders, local officials, teachers, pharmacy owners, midwives and pregnant women	50 ^ab^
Mbonye—UGA [34]	2006 (2002–2003)	Perceptions about SP-IPTp	Adolescent (10–19 years), young (20–29 years) and adult (30–49 years) women, opinion leaders, local officials, elderly midwives, retired teachers, pharmacy owners, midwives and pregnant women	50 ^ab2^
Mbonye—UGA [35]	2006 (2002–2003)	Perceptions about MIP. Signs of pregnancy and malaria that lead to the search for treatment	Opinion leaders (local council officials, elderly midwives, retired teachers, pharmacy owners), midwives, and pregnant and non-pregnant women and men	42 ^ab2^
Ahorlu—GHA [36]	2007 (2002–2004)	Importance of culture and behavior in malaria transmission, prevention and control	Men and women	67 ^a3^
Launiala—MWI [37]	2007 (2002)	Factors affecting compliance with the SP-IPTp Scheme	Pregnant women	92 ^ac3^
Mubyazi—TZA [38]	2008 (2006–2007)	Perspectives, achievements, challenges and opportunities to implement SP-IPTp	Officials at the national level	75 ^b1^
Brabin—GMB [39]	2009 (2007)	SP-IPTp awareness	Young and older married women, adolescents, midwives and men	67 ^a^
Chukwuocha—NGA [40]	2010 (No data)	Perceptions about ITN to prevent MiP	Adolescents, young women and men, opinion leaders, local government officials, elderly midwives, retired leaders, pharmacy owners, midwives, pregnant and non-pregnant women.	25 ^ab^
Mubyazi—TZA [41]	2010 (2005–2006)	Perceptions and attitudes about barriers and costs of SP-IPTp and other ANC services	Pregnant women and mothers of young children	67 ^abc1^
Smith—GHA [42]	2010 (2009)	Acceptability, experiences and perceptions of ANC and clinical trial participation	Women enrolled in a clinical trial	50 ^ab2^
Onoka—NGA [43]	2012 (2010)	Provider Factors Affecting SP-IPTp Delivery	Public and private ANC	42 ^b^
Diala—NGA [44]	2013 (2012)	Perceptions about SP-IPTp and barriers to adherence	Pregnant women who attend ANC and others who do not attend, with their husbands	58 ^a1^
Menaca—GHA/KEN/MWI [45]	2013 (2009–2011)	Local understanding of MiP	Pregnant women, relatives, opinion leaders, health providers and community members	75 ^ac^
Pell—GHA/KEN/MWI [46]	2013 (2009–2011)	Sociocultural factors related to the prevention and management of MiP	Pregnant women, relatives, opinion leaders, health providers, community members	75 ^ac^
Webster—MLI [47]	2013 (No data)	Factors that explain ineffectiveness of SP-IPTp and ITN	HWs at the national, regional, district and health facility levels	58 ^b^
Dræbela—SDN [48]	2014 (2008)	Perceptions and practices of prevention and treatment of MiP	Pregnant women	33 ^b^
Pell—GHA [49]	2014 (2010)	Acceptability of screening and intermittent treatment artemether-lumefantrine compared to SP-IPTp	Pregnant women and staff of the clinical trial	50 ^ac2^
Andrew—PNG [50]	2015 (2010–2011)	KAP on MiP and its prevention	Pregnant women, HWs, community members, relatives (husbands, parents, siblings)	83 ^ac^
Hill—KEN/MLI [51]	2015 (2009–2010)	Operational, socioeconomic and cultural barriers to access and use IPTp, ITN and case management	Men, adolescents, not pregnant and pregnant	58 ^ab^
Onyeneho—NGA [52]	2015 (No data)	Perceptions and attitudes about MiP prevention	Mothers and husbands of women who gave birth within 6 months, HWs and women who gave birth within 6 months	75 ^a1^
Yoder—MWI [53]	2015 (2013)	Experiences in the provision of ANC and SP-IPTp services	ANC suppliers	67
Hill—KEN [54]	2016 (2013–2014)	Acceptability of intermittent screening and treatment with dihydroartemisinin-piperaquine	Pregnant women and HWs	83 ^a2^
Jaiteh—GMB [55]	2016 (2014)	Perceptions of MiP and its influence on adherence to treatment	Community nurses, husbands, mothers-in-law, women of reproductive age, community HWs, midwives	83 ^ac23^
Klein—MLI [56]	2016 (2013–2014)	Perceptions, barriers and experiences of the cost of SP-IPTp	Pregnant women, HWs and people from the community	75 ^ac^
Rassi—UGA [57]	2016 (2013–2014)	Barriers in the demand for SP-IPTp (access, affordability and acceptability)	District health officials, HWs, pregnant women and opinion leaders	83 ^b1^
Rassi—UGA [58]	2016 (2013–2014)	Barriers in the supply of SP-IPTp	District health officials, HWs	83 ^b^
Manu—GHA [59]	2017 (2010)	Factors associated with ITN use	Pregnant women	67 ^abc1^
Quist—GHA [60]	2017 (2015)	KAP on the use of ITN	Pregnant women	58 ^b^
Hill—IDN [61]	2018 (2015)	Acceptability and perceptions of the feasibility of a single screening and treatment strategy	ANC providers (midwives, physicians, laboratory personnel, pharmacists and pharmacy managers), heads of health establishments and staff of the District Health Office.	50 ^b2^
Hoyt—IDN [62]	2018 (2015–2016)	Acceptability of “one-off screen and treat” strategy compared to SP-IPTp, and intermittent screen-and-treat	ANC providers (midwives, physicians, laboratory personnel, pharmacists, pharmacy manager), heads of health establishments and staff of the District Health Office, and pregnant women	58 ^ab2^
Martínez—LBR [63]	2018 (2016–2017)	Barriers to participate in research on MiP	Hospital staff, representatives of traditional communities and pregnant women	92 ^a4^
Sabin—IND [64]	2018 (2007–2008)	KAP on prevention and treatment of MiP	Pregnant women and HWs	75
Tarr—LBR [65]	2018 (2016–2017)	Knowledge about etiology, prevention and therapeutics of MiP. Perceptions of the usefulness of MiP research	Pregnant women, community leaders and hospital staff	75 ^a4^
Aberese—GHA [66]	2019 (2018–2019)	Health system, sociocultural, economic, environmental, and individual factors influencing ITN ownership and use	HWs, pregnant women and community members	83 ^bc3^
Arnaldo—MOZ [67]	2019 (2015)	Factors that limit the access and use of IPTp-SP in a rural area	Pregnant women and nurses	67 ^b^
Aberese—GHA [68]	2020 (2018–2019)	Challenges for the implementation of MiP policies, consequences in the adoption of SP-IPTp and access to maternal health care	HWs, pregnant women and community members	92 ^b3^
Enguita—NGA/MOZ/COD/MDG [69]	2020	Factors influencing the acceptability of SP-IPTp	Pregnant women, community leaders, relatives, HWs, formal and informal health providers	92 ^a4^
Palmer—GHA [70]	2020 (2015)	Facilitators, barriers and use of tests at the point-of-care for MiP	Staff and pregnant women	67 ^a^
Aberese—GHA [71]	2021 (2018–2019)	Health system, sociocultural and individual factors influencing the adoption of SP-IPTp	HWs, pregnant women and community members	92 ^bc3^
Burke—BFA [72]	2021 (2017–2018)	Perceptions about two SP-IPTp delivery modalities	Community HWs and clinical facilities	58
Faye—NGA/MOZ/COD [73]	2021 (2018)	Perception and acceptability of SP-IPTp, new SP packaging and communication tools for its use	Health care providers, community HWs, and pregnant women	75 ^ac5^
Hoyt—KEN [74]	2021 (2015)	Perceptions about intermittent preventive treatment, screening and treatment with dihydroartemisinin-piperaquine	Pregnant women and HWs	58 ^ab^
Muhammad—NGA [75]	2021	Barriers to the use of SP-IPTp	Malaria experts, program coordinators, community HWs and pregnant women	83 ^ab^
Nyaaba—NGA [76]	2021 (2019)	Factors associated with low acceptance of SP-IPTp and ITN	Public health care providers, midwives, community leaders, caregivers, relatives, pregnant women	83 ^4^
Osarfo—GHA [77]	2021 (2012)	Experiences and perceptions about participation in a clinical trial	Pregnant women and husbands	42 ^2^
De Gaukke—GHA [78]	2022 (2019)	Contextual factors of the health system that influence the delivery of SP-IPTp and ITN	HWs and administrative staff	83 ^a^
Taremwa—UGA [79]	2022 (2020)	Factors that influence the prevention of MiP and the impact of COVID-19	Pregnant women, midwives, village health teams, local leaders, and healthcare providers	75

* Publication (conducting the study). ^a^ Focus group discussion. ^b^ Thematic or content analysis. ^c^ Observation. ^1^ Added to cross-sectional study. ^2^ Nested to community intervention or clinical trial. ^3^ Ethnography. ^4^ Grounded theory. ^5^ Participatory research.

**Table 2:** Categorial system according to the research topic.

Category	Subcategories	Properties
Antenatal Care		
Positive perceptions	Health benefits	(i) health control and prevention, (ii) diagnostic tests, (iv) drugs and vitamins
	Type of care	(i) courtesy–patience, (ii) trust, (iii) home visits, (iv) free of charge
Experiences that prevent use	Costs	(i) transportation to clinic, (ii) wasting time, (iii) user fees and unofficial payments
	Negative perception	(i) due to gender roles and rurality, (ii) by low social support, (iii) negative attitude of the HW, (iv) refusal to take medicine or attend appointments
Malaria in Pregnancy		
Knowledge attitudes and practices	Local understanding and knowledge of the disease	(i) overlapping of local and biomedical concepts, (ii) multiple causes of abortion, anemia and low birth weight, (iii) low knowledge on etiology, preventive methods with proven efficacy, and treatment of MiP
	Attitudes (perceptions) and practices (behaviors)	(i) positive attitude to seek treatment in symptomatic cases, (ii) low perception of MiP risk, (iii) regular perception of preventive methods, (iv) mistrust due to drug effects, (v) high trust in and use of traditional medicine
Determinants of prevention	Sociocultural determinants	(i) local worldviews and trust in traditional medicine, (ii) low education, poverty and economic dependence
	Determinants of the health system	(i) shortages and rationing, (ii) delay in payments and reimbursement to hospitals, (iii) out-of-pocket health expenses
Insecticide Treated Net		
Knowledge attitudes and barriers of its acceptability	Knowledge and attitudes	(i) High knowledge on benefits, (ii) negative attitudes towards its use, (iii) preference for traditional mosquito management
	Sociocultural barriers	(i) erroneous beliefs, (vii) family and gender roles
	Health system barriers	(i) ITN cost, (ii) shortages, (iii) governance, financing and human resource problems, (iv) low knowledge and negative attitude of HW
Intermittent Preventive Treatment in Pregnancy with Sulfadoxine-pyrimethamine		
Knowledge attitudes and practices	Knowledge and attitudes	(i) low knowledge in pregnant women, (ii) HWs with high knowledge about effectiveness but moderate knowledge about implementation, (iii) positive perception of effectiveness in pregnant women and HWs, (iv) pregnant women distrust of effects on maternal–fetal health (negative attitudes about their safety)
	Practices	(i) low adherence, (ii) preference of HWs for observed treatment
Determinants of acceptability	Individual determinants	(i) late admission to ANC, (ii) low understanding of the intervention, (iii) refusal to take SP or resistance to pharmacological measures for prevention
	Healthcare system	(i) quality of health care, (ii) information of HW, (iii) work overload, (iv) shortages, (iv) costs and out-of-pocket expenses
Case Detection and Management		
Acceptability	Attitudes	(i) negative attitudes about the diagnosis and the efficacy of the treatment, (i) negative attitudes about the adverse effects of the treatment
	Barriers	(i) distrust of HW in rapid tests and safety of some drugs, (ii) adherence problems, (iii) distance to clinic, (iv) low availability of tests and drugs

Note: One topic was determined that was not included in the table since it only had one category: Utility or benefits of participating in research on MiP.

## Data Availability

Not applicable.
